# Identification of potassium and calcium channel inhibitors as modulators of polyomavirus endosomal trafficking

**DOI:** 10.1016/j.antiviral.2020.104819

**Published:** 2020-07

**Authors:** Samuel J. Dobson, Jamel Mankouri, Adrian Whitehouse

**Affiliations:** a School of Molecular and Cellular Biology, Faculty of Biological Sciences, United Kingdom; b Astbury Centre for Structural Molecular Biology, University of Leeds, Leeds, United Kingdom

**Keywords:** Polyomavirus, Ion channels, Two pore channel, Endosomal fusion, Verapamil, Tetrandrine

## Abstract

During virus entry, members of the *Polyomaviridae* transit the endolysosomal network *en route* to the endoplasmic reticulum (ER), from which degraded capsids escape into the cytoplasm and enter the nucleus. Emerging evidence suggests that viruses require both endosomal acidification and the correct ionic balance of K^+^ and Ca^2+^ ions in endosomes for correct virus trafficking and genome release. Here, using two polyomaviruses with different capsid architectures, namely Simian virus 40 (SV40) and Merkel cell polyomavirus (MCPyV), we describe methods to rapidly quantify virus infection using IncuCyte ZOOM imaging analysis, and use this system to investigate the role of both K^+^ and Ca^2+^ channels during the early stages of virus entry. Using broad spectrum blockers of both K^+^ and Ca^2+^ channels to specifically target host cell ion channel functionality, we show that MCPyV, but not SV40 can be inhibited by K^+^ channel modulators, whilst both viruses are restricted by the broad spectrum Ca^2+^ channel inhibitor verapamil. Using a panel of more specific Ca^2+^ blockers, we show that both MCPyV and SV40 are dependent on the activity of two-pore Ca^2+^ channels (TPCs), as the TPC-specific blocker tetrandrine prevented capsid disassembly and nuclear transport required for virus entry. We therefore reveal a novel target to restrict the entry of polyomaviruses, which given the known role of TPCs during endolysosomal-ER fusion, is likely to be applicable to other viruses that transit this pathway.

## Highlights

• We describe novel high-throughput assays to study SV40 and MCPyV infection.
• MCPyV, but not SV40, is sensitive to K^+^ channel inhibition.
• Verapamil inhibits MCPyV and SV40 infection.
• Tetrandrine is a potent inhibitor of MCPyV and SV40 infection.
• Two-pore channel 1/2 activity is essential for polyomavirus entry.

## 1 Introduction

Polyomaviruses (PyVs) are small double stranded DNA viruses that establish persistent infections in their hosts. Whilst human PyV infections are generally asymptomatic, they can cause severe disease particularly in the immunosuppressed. Common examples include BKPyV-associated nephropathy and haemorrhagic cystitis, JCPyV-induced progressive multifocal leukoencephalopathy (PML) and MCPyV-positive Merkel cell carcinoma (Feng et al., 2008; Gardner et al., 1971; Knowles, 2006; Padgett et al., 1971). Current therapies to treat PyV-induced diseases are limited, therefore there is a need to develop new strategies.

The capsids of all PyVs consist of 72 VP1 pentamers that form an icosahedral structure with T = 7d symmetry and mediate initial surface receptor binding (Hurdiss et al., 2016; Moens et al., 2017; Neu et al., 2010). Under each pentamer sits a minor capsid protein linking VP1 to the viral genome (Hurdiss et al., 2016). The majority of PyVs, including SV40, BKPyV and JCPyV encode two minor capsid proteins (VP2 and VP3) which are incorporated into the capsid. MCPyV is however part of a small clade of PyVs that express only one minor capsid protein (VP2) (Schowalter and Buck, 2013).

All PyVs must deliver their genomes to the nucleus, commonly achieved by trafficking through the endosomal system (Qian et al., 2009; Tsai and Qian, 2010). Initial attachment varies across PyV species but typically involves sialylated glycans. SV40 interacts with MHC-1 and GM1 gangliosides in lipid rafts, whilst MCPyV interacts with sulphated glycosaminoglycans including heparan sulphate or chondroitin sulphate prior to secondary interactions with sialylated glycans to facilitate virus penetration (Anderson et al., 1998; Clayson et al., 1989; Miller-Podraza et al., 1982; Schowalter et al., 2011; Stang et al., 1997). Following binding, JCPyV enters cells through clathrin-mediated endocytosis, whilst SV40, MCPyV and BKPyV enter via caveolar/lipid rafts (Becker et al., 2019; Eash et al., 2004; Gilbert and Benjamin, 2000; Mayberry et al., 2019; Moriyama et al., 2007; Pho et al., 2000). Virions traffic through the endosomal system and in response to endosomal cues, including endosome acidification, initiate proteolytic rearrangements of the capsid prior to retrograde trafficking to the endoplasmic reticulum (ER) (Becker et al., 2019; Engel et al., 2011; Kuksin and Norkin, 2012; Mercer et al., 2010). Within the ER, virions are further disassembled, exposing nuclear localisation signals (NLSs) that transport capsids to the nucleus via importins (Geiger et al., 2011; Nakanishi et al., 2007, 2002; Nishikawa et al., 2001; Pelkmans et al., 2001; Schelhaas et al., 2007; Yamada and Kasamatsu, 1993). Despite this knowledge, the endosomal cues that permit PyV trafficking remain poorly understood.

Emerging studies suggest that the current description of virus entry processes involving acidification alone are too simplistic and that the accumulation of other ions including K^+^ and Ca^2+^ influence virus trafficking (Dubey et al., 2019; Gehring et al., 2014; Hover et al., 2018, 2017; 2016; Sakurai et al., 2015). In the context of PyV infection, Ca^2+^ ions have been shown to affect the structure and organisation of virus particles, regulating their disassembly through virion swelling (Asor et al., 2019; Hover et al., 2018; Ishizu et al., 2001; Li et al., 2003). However, despite the evidence that cellular ion channels are targeted by a wide range of viruses to enhance specific lifecycle stages, their role during PyV entry has not been defined (Choi et al., 2008; Dubey et al., 2019; Evans et al., 2015; Gehring et al., 2014; Herrmann et al., 2010; Hover et al., 2018; Igloi et al., 2015; Mankouri et al., 2009; Sakurai et al., 2015; Stakaityte et al., 2018; Zheng et al., 2014). Given the vast array of potent small molecule inhibitors targeting ion channels in clinical use, repurposing of approved drugs may present novel therapeutic options to restrict PyV infections.

In this study, we used two distantly related PyVs, namely SV40 and MCPyV, to determine if K^+^ or Ca^2+^ channels are required for viral progression through the endosomal system. To achieve this, reporter-containing MCPyV pseudovirions (PsVs) that behave in a similar manner to WT viruses were used alongside native SV40 virions to specifically assess virus entry through high-throughput fluorescence-based detection systems. Herein, we show that MCPyV and SV40 are differentially sensitive to K^+^ channels and transient (T-type) Ca^2+^ channel inhibitors. We further identify a shared requirement of SV40 and MCPyV for the activity of endosomal nicotinic acid adenine dinucleotide phosphate (NAADP)-Sensitive Two-Pore Ca^2+^ Channels (TPCs) that regulate ER-endosome membrane contact sites. These findings reveal potential therapeutic drug targets for PyVs and enhance our understanding of the virus entry processes.

## 2 Materials and methods

### 2.1 Antibodies and chemicals

The (pAb)108 hybridoma used to detect SV40 T-antigens was a kind gift from Daniel DiMaio (Yale Cancer Centre, Connecticut, USA). SV40 VP2/3 and calnexin antibodies were purchased from Abcam and Thermo Fisher Scientific, respectively.

EGA, gabapentin, KCl, quinine HCl, TEA, tetrandrine and verapamil were purchased from Sigma-Aldrich. 4AP and flunarizine were purchased from Alfa Aesar. Nitrendipine and NH_4_Cl were purchased from Santa Cruz Biotech.

### 2.2 Cell lines and maintenance

HEK293TT (293TT) cells were a kind gift from Christopher Buck (NIH, National Cancer Institute, Bethesda, MD, USA). Vero cells were a kind gift from Andrew Macdonald (University of Leeds, Leeds, UK). Cells were maintained using Dulbecco's modified Eagle's medium (DMEM) containing 10% (v/v) foetal bovine serum (FBS) and 50 U/mL penicillin and streptomycin (complete DMEM). 293TT medium was supplemented with 250 μg hygromycin B (Thermo Fisher Scientific) to maintain T-antigen expression, with removal prior to experimentation.

### 2.3 SV40 production and titration

SV40 stocks were produced through infection of naïve Vero cells, with virus progeny containing medium removed 7 days post infection.

To determine virus stocks, dilutions were incubated on cells in triplicate for 2 h before aspiration and addition of fresh complete DMEM. Cells were fixed 24 h post infection (hpi) and SV40 T-antigens were detected using (pAb)108 and species-specific Alexa Fluor 488 (Life Technologies, Thermo Fisher Scientific) antibodies. Wells were imaged using an Incucyte ZOOM instrument, with 4 non-overlapping images taken in each well. The number of T-antigen positive cells were counted per well. Reciprocals were calculated to identify dilutions with a linear relationship between dilution and T-antigen positive cells. Values were used to calculate IU/mL.

### 2.4 SV40 infection assay

Vero cells were pre-treated with chemical inhibitors for 1 h prior to infection with SV40 at an MOI of 1 relative to initial seeding. Cells were fixed 24 hpi, immunostained, imaged and analysed using an Incucyte ZOOM System as described above. Inhibitor effects were calculated through comparison to untreated controls. To evaluate potential cytotoxicity of chemical inhibitors, percentage confluence was determined by Incucyte ZOOM analysis to ensure continued proliferation in comparison to untreated cells. Chemical inhibitors calculated to display <80% comparable confluence were deemed to be cytotoxic and omitted. Error bars represent the standard deviation of three experimental repeats, where four images per well were used to calculate the number of LT-positive cells per well.

### 2.5 MCPyV PsV production

Production of MCPyV PsVs has been previously described (Buck and Thompson, 2007; Pastrana et al., 2009; Schowalter et al., 2011; Schowalter and Buck, 2013). Briefly, 293TT cells were transfected with pwM2m, ph2m and pEGFP-C1 and harvested by trypsinisation 48 h post transfection before lysis and overnight maturation. PsVs were extracted by centrifugation and loaded onto a 27-33-39% discontinuous opti-Prep (Sigma-Aldrich) gradients prior to ultracentrifugation. Fractions were collected and samples were analysed by Western blotting and silver staining to identify PsV-containing fractions which were pooled. BSA standards were separated by SDS-PAGE alongside gradient fractions prior to silver staining to determine relative mass of PsVs in each fraction.

### 2.6 MCPyV reporter assays

If required, pre-treatment with chemical inhibitors was performed for 1 h prior to addition of 10 ng VP1 equivalent of MCPyV PsV stock diluted in complete DMEM (containing chemical inhibitor if required) and added to wells of a 24 well plate containing 293TT cells for 2 h. 72 h post transduction (hpt) detection of GFP positive cells was performed using an Incucyte ZOOM System. Chemical inhibitor effects were calculated through comparison to an untreated control. To evaluate potential cytotoxicity of chemical inhibitors, percentage confluence was determined by Incucyte ZOOM analysis to ensure continued proliferation in comparison to untreated cells. Chemical inhibitors displaying <80% comparable confluence were deemed to be cytotoxic and omitted. Error bars represent the standard deviation of three experimental repeats, where twelve non-overlapping images per well were used to calculate the number of GFP-positive cells per well.

### 2.7 Minor capsid protein exposure assay

Plates were chilled at 4 °C for 30 min before addition of SV40 virions at an MOI of 3 in pre-chilled complete DMEM (containing inhibitors if applicable). Cells were kept at 4 °C for 1 h with gentle agitation every 15 min to permit virus binding before addition of pre-warmed complete DMEM (containing inhibitors if applicable) to synchronise infection. Cells were fixed 10 hpi and immunofluorescence microscopy performed as previously described (Schumann et al., 2016). VP2/3 specific antibodies were used to detect exposed minor capsid proteins, with calnexin antibodies used to visualise proximity to the ER. Microscopy was performed using a ZEISS LSM 880 confocal microscope.

### 2.8 Statistical analysis

Student's *t*-tests were performed to determine statistical significance compared to untreated controls, with *P*-values assigned whereby ** = ≤0.005, *** = ≤0.0005 and **** = ≤0.0001.

## 3 Results

### 3.1 Fluorescence-based detection of SV40 and MCPyV

**Fig. 1:**
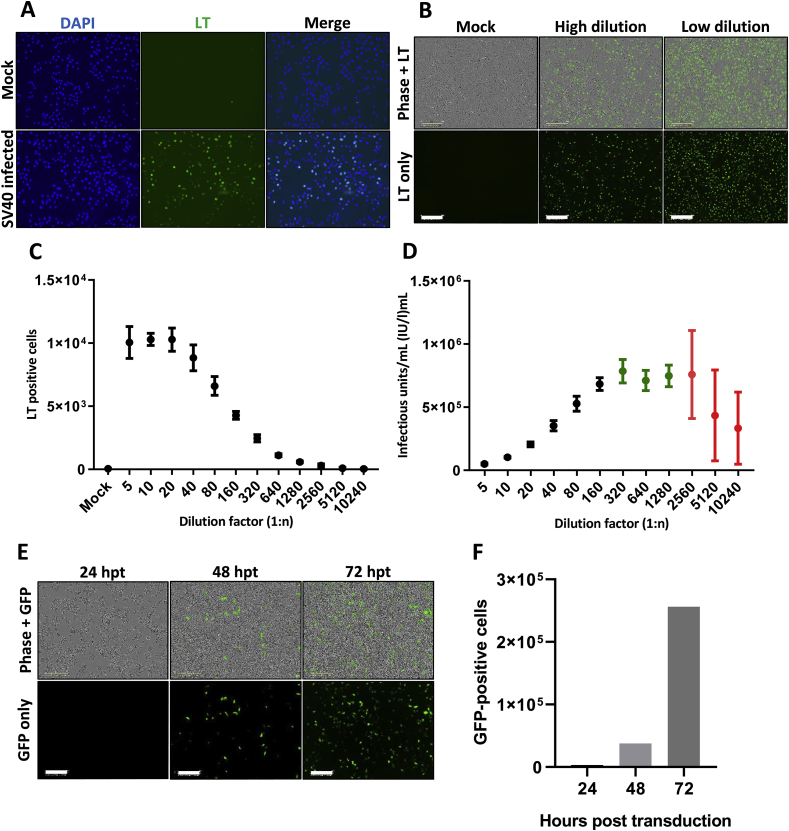
Development of immunofluorescence-based systems to determine SV40 titre and study early events in the lifecycles of SV40 and MCPyV. Visualisation of SV40 infected Vero cells 24 h post infection by light microscopy (**A**) and Incucyte ZOOM instrument (**B**). For light microscopy DAPI was used to stain nucleic acids whilst SV40 LT/ST specific primary and Alexa Fluor 488 secondary antibodies were used to visualise infected cells. Images were taken using an EVOS II microscope. For validation by Incucyte detection, SV40 infected Vero cells were similarly immunostained before imaging. Shown are representative images using differentially diluted virus stock, scale bar 300 μM. (**C**) SV40 T-antigen positive cell counts were determined by Incucyte ZOOM detection at a range of dilutions. Reversal of dilution factors was performed to determine infectious units/mL (**D**), whereby the plateau represented viral titre (green) and hypervariable replicates were indicative of loss of assay sensitivity (red). (**E**) Viability of Incucyte detection for GFP-expressing MCPyV PsVs was also confirmed by imaging at 24-, 48- and 72-h post transduction, with autonomous quantification of GFP-positive cells (**F**). Scale bar 300 μM.

A challenge in PyV studies are the limited experimental systems to assess the complete virus lifecycle. We therefore focussed on developing high throughput assays to permit antiviral screening of early events. Initially, we established a fluorescence detection system to monitor the expression of SV40 T-antigens following virus infection using an Incucyte ZOOM instrument, in a manner comparable to previous methods described for Hepatitis C virus measurement (Fig. 1A and B) (Charlton et al., 2019; Stewart et al., 2015). The system could reproducibly quantify the number of SV40 T-antigen positive cells and could be used as a rapid method to assess the levels of virus infection (Fig. 1C and D). Whilst an infectious system has previously been reported using dermal fibroblasts, the study of MCPyV remains more challenging as such systems require extensive manipulation through supplement of chemical inhibitors (Liu et al., 2016). We therefore applied reporter-containing PsVs that permit the assessment of MCPyV entry and genome release into the nucleus. MCPyV PsVs transduced target cells more slowly than SV40 infection, with detectable fluorescence observed between 48 and 72 hpt, consistent with previously reported timescales (Fig. 1E and F) (Becker et al., 2019). Using these systems, SV40 infections and MCPyV transductions could be performed in a 96- or 24-well plate format, respectively, providing a platform for high-throughput antiviral compound screening.

**Fig. 2:**
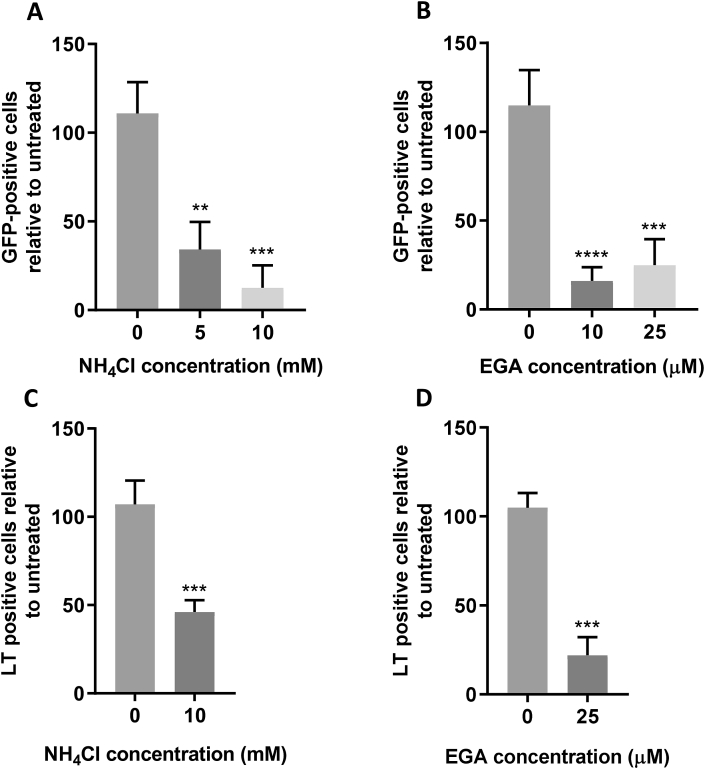
MCPyV and SV40 both enter into acidified endosomes. (**AandB**) 293TT cells were incubated with drug as described for 1 h before addition of 10 ng VP1-equivalent MCPyV GFP PsVs for 2 h with occasional agitation. PsV containing medium was removed and replaced with fresh drug-containing medium, with Incucyte detection 72 h post transduction to determine the number of GFP-positive cells. (**CandD**) Vero cells were incubated with drug as described for 1 h before addition of SV40 virions at an MOI of 1 for 2 h with occasional agitation. Fresh drug-containing medium was then added for an incubation of 24 h before fixation and permeabilisation. SV40 T-antigens were immunostained using an SV40 LT/ST antibody and species-specific Alexa Fluor 488 secondary antibody. Wells were then imaged using an Incucyte ZOOM instrument to determine the number of T-antigen positive cells, which are presented as a percentage relative to an untreated control. A relative volume of H_2_O or DMSO vehicle control was used for 0 mM NH_4_Cl and 0 μM EGA, respectively.

It is well established that PyVs traffic through the endolysosomal system where acidification initiates proteolytic rearrangements to promote virus disassembly. To validate our Incucyte-based system, we assessed virus infection in the presence of ammonium chloride (NH_4_Cl), a known inhibitor of endosomal acidification. Consistent with previous studies, NH_4_Cl treatment reduced MCPyV and SV40 infection by 87% and 54% respectively, further validating the system (Fig. 2A and C) (Becker et al., 2019; Engel et al., 2011).

Despite knowledge that acidification is important, the endosomal progression of PyVs prior to ER translocation remain unclear. To determine whether the viruses enter late endosomes and/or lysosomes, cells were treated with 2-[(4-Bromophenyl) methylene]-N-(2, 6-dimethylphenyl)-hydrazinecarboxamide (EGA) to inhibit lysosomal clustering prior to virus infection. Treatment with EGA led to a 75% and 78% decrease in MCPyV and SV40 infected cells respectively, suggesting that both viruses transverse the late endolysosomal system (Fig. 2B and D).

### 3.2 K^+^ and Ca^2+^ channel inhibition restricts MCPyV entry

**Fig. 3:**
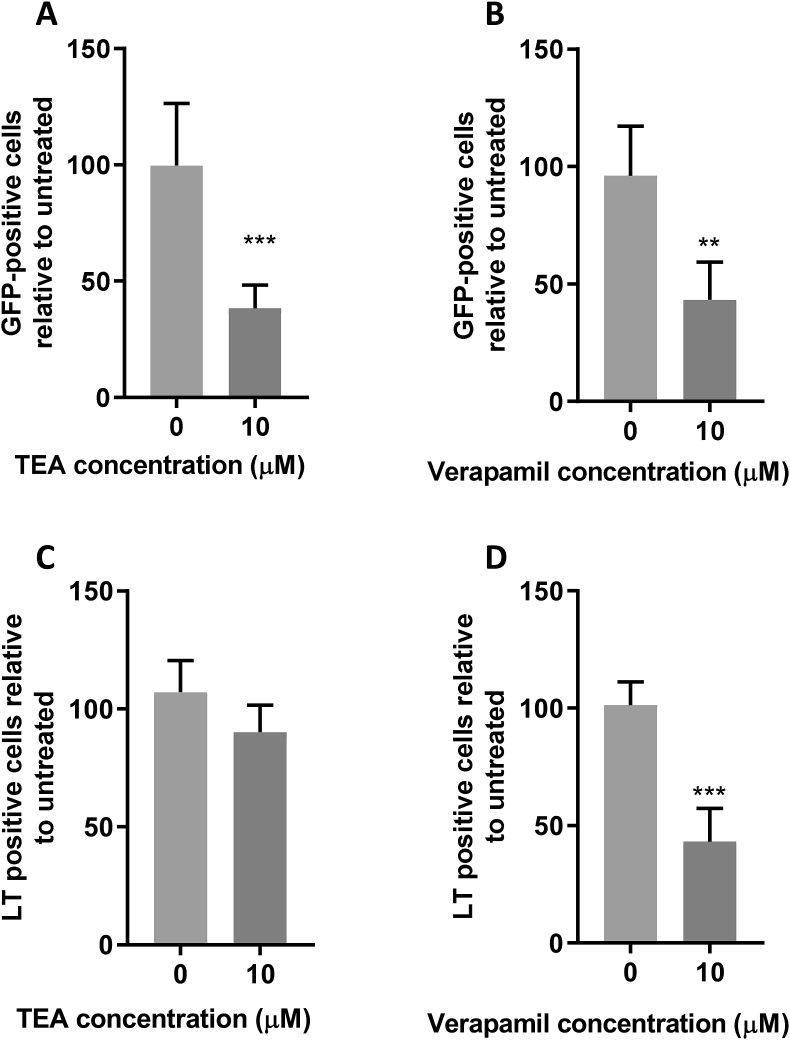
MCPyV and SV40 have a conserved requirement of Ca^2+^ channels, whilst only MCPyV requires K^+^ channel activity. (**AandB**) 293TT cells were incubated with drug as described for 1 h before addition of 10 ng VP1-equivalent MCPyV GFP PsVs for 2 h with occasional agitation. PsV containing medium was removed and replaced with fresh drug-containing medium, with Incucyte detection 72-h post transduction to determine the number of GFP-positive cells. (**CandD**) Vero cells were incubated with drug as described for 1 h before addition of SV40 virions at an MOI of 1 for 2 h with occasional agitation. Fresh drug-containing medium was then added for an incubation of 24 h before fixation and permeabilisation. SV40 T-antigens were immunostained using an SV40 LT/ST antibody and species-specific Alexa Fluor 488 secondary antibody. Wells were then imaged using an Incucyte ZOOM instrument to determine the number of T-antigen positive cells, which are presented as a percentage relative to an untreated control. A relative volume of vehicle (H_2_O) was used for 0 μM controls.

Ion channels have emerged as key regulators of virus entry processes. Examples include the negative sense RNA viruses, bunyamwera virus and influenza virus that require K^+^ during endosomal transit to mediate virus priming and endosomal escape (Hover et al., 2018, 2016; Stauffer et al., 2014). We therefore explored whether regulators of two of the major endosomal ion channel families, namely K^+^ or Ca^2+^ channels, were important for PyV entry. We selected a pharmacological approach to specifically target channel functionality since gene-silencing approaches can ablate other cellular roles of the ion channel proteins. Treatment of cells with the broad spectrum K^+^ channel inhibitor tetraethylammonium (TEA) and broad-spectrum Ca^2+^ channel inhibitor verapamil inhibited MCPyV infection by 62% and 57%, respectively, suggesting that both channel families were important during MCPyV entry (Fig. 3A and B). In contrast, TEA had little to no-effect on SV40 infection (Fig. 3C), whereas Verapamil treatment caused a 57% reduction in SV40 infection (Fig. 3D), suggesting a conserved requirement of Ca^2+^ channels.

### 3.3 Identification of the K^+^ channels required during MCPyV entry

**Fig. 4:**
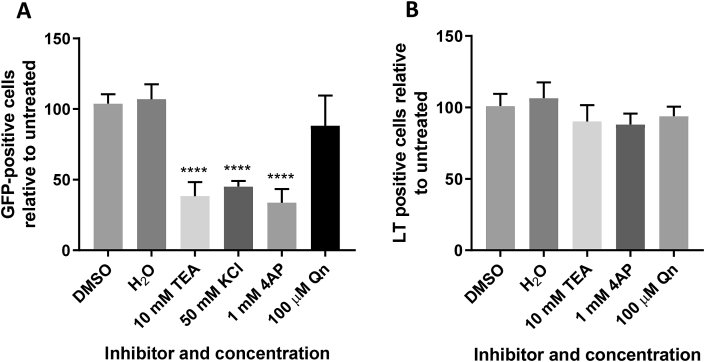
Requirement of K^+^ channel activity during entry is MCPyV specific. (**A**) 293TT cells were incubated with drug as described for 1 h before addition of 10 ng VP1-equivalent MCPyV GFP PsVs for 2 h with occasional agitation. PsV containing medium was removed and replaced with fresh drug-containing medium, with Incucyte detection 72 h post transduction to determine the number of GFP-positive cells. (**B**) Vero cells were incubated with drug as described for 1 h before addition of SV40 virions at an MOI of 1 for 2 h with occasional agitation. Fresh drug-containing medium was then added for an incubation of 24 h before fixation and permeabilisation. SV40 T-antigens were immunostained using an SV40 LT/ST antibody and species-specific Alexa Fluor 488 secondary antibody. Wells were then imaged using an Incucyte ZOOM instrument to determine the number of T-antigen positive cells, which are presented as a percentage relative to an untreated control. 50 mM KCl was omitted due to Vero cell cytotoxicity.

K^+^ channels are the most diverse class of membrane proteins expressed with the cell (Grizel et al., 2014). There are four subfamilies that are ubiquitously expressed across nearly all kingdoms of life: (I) voltage-gated K^+^ channels (K_V_) (6 transmembrane domains (TMDs)), (II) inwardly rectifying K^+^ channels (K_IR_) (2 TMDs), (III) tandem pore domain K^+^ channels (K_2P_) (4 TMDs) and Ca^2+^ activated K^+^ channels (K_Ca_) (6 TMDs) (Salkoff, 2006). To identify which K^+^ channel subfamilies are required during MCPyV entry, we investigated the effects of KCl (to destroy K^+^ gradients and thus K^+^ channel function) and 4-aminopyridine (4AP, a K_V_ channel blocker) on MCPyV and SV40 infection. Both KCl and 4AP inhibited MCPyV, however 4AP had no effect upon SV40 (Fig. 4A and B). Furthermore, the anti-malarial drug quinine that promiscuously blocks a variety of K^+^ channels through an unknown mechanism had no effect on either MCPyV or SV40. These data highlighted differences in MCPyV and SV40 entry processes and suggested that MCPyV can be blocked by inhibitors of 4AP sensitive, quinine insensitive Kv channels.

### 3.4 Blockers of L-type Ca2^+^ channels restrict MCPyV entry

**Fig. 5:**
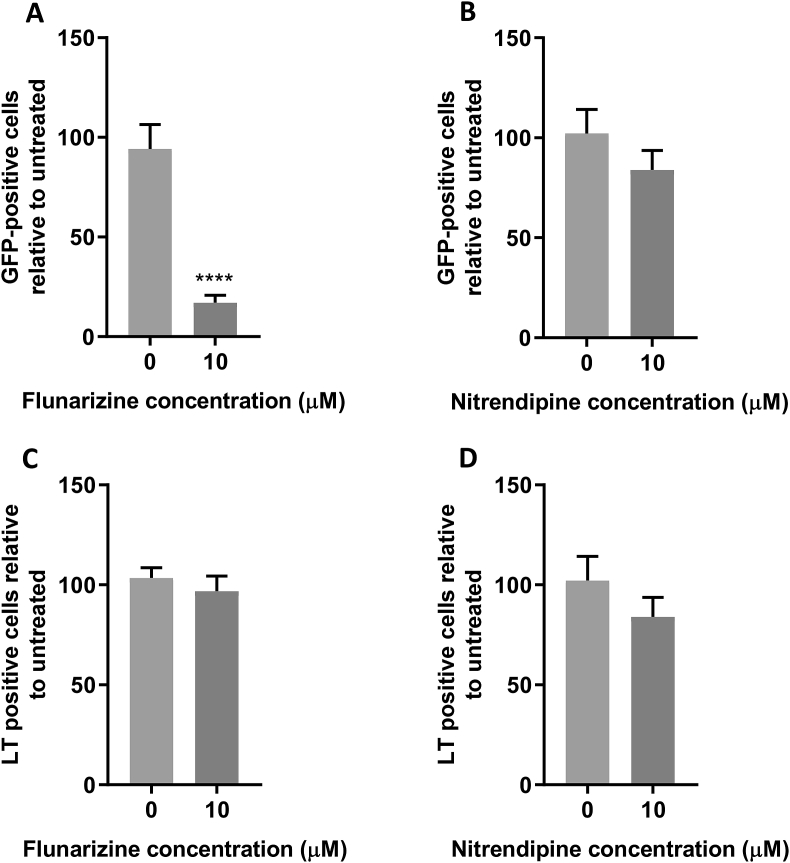
Inhibition of T-type Ca^2+^ channels restricts MCPyV entry, but SV40 is not affected by inhibition of T- or L-type Ca^2+^ channels. (**AandB**) 293TT cells were incubated with drug as described for 1 h before addition of 10 ng VP1-equivalent MCPyV GFP PsVs for 2 h with occasional agitation. PsV containing medium was removed and replaced with fresh drug-containing medium, with Incucyte detection 72 h post transduction to determine the number of GFP-positive cells. (**CandD**) Vero cells were incubated with drug as described for 1 h before addition of SV40 virions at an MOI of 1 for 2 h with occasional agitation. Fresh drug-containing medium was then added for an incubation of 24 h before fixation and permeabilisation. SV40 T-antigens were immunostained using an SV40 LT/ST antibody and species-specific Alexa Fluor 488 secondary antibody. Wells were then imaged using an Incucyte ZOOM instrument to determine the number of T-antigen positive cells, which are presented as a percentage relative to an untreated control.

Given that K^+^ channel inhibition did not display a conserved effect upon MCPyV and SV40 entry, the effect of verapamil was further investigated. Verapamil inhibits both Transient (T-type, low-voltage activated) and long lasting (L-type, high-voltage activated) Ca^2+^ channel family members, and as such, more specific Ca^2+^ blocking drugs were assessed for their effects on SV40 and MCPyV. Treatment with the T-type inhibitor flunarizine led to an 83% inhibition of MCPyV infection, whilst nitrendipine (an L-type Ca^2+^ channel blocker) had no significant effect (Fig. 5A and B). Both flunarizine and nitrendipine did not affect SV40 entry suggesting that the requirement for T-type Ca^2+^ channels is limited to MCPyV (Fig. 5C and D).

### 3.5 Blockers of two pore Ca^2^^+^ channels inhibit MCPyV and SV40

**Fig. 6:**
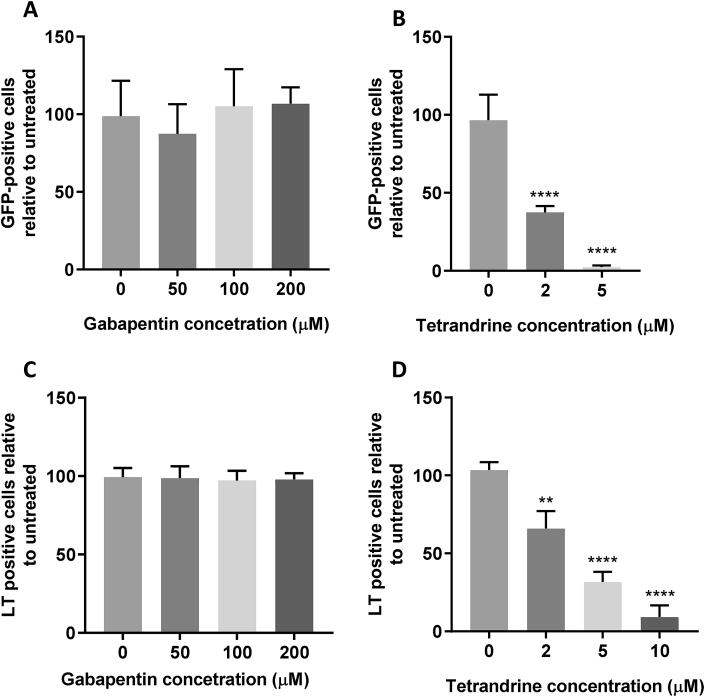
Two pore channel activity is essential for MCPyV and SV40 entry. (**AandB**) 293TT cells were incubated with drug as described for 1 h before addition of 10 ng VP1-equivalent MCPyV GFP PsVs for 2 h with occasional agitation. PsV containing medium was removed and replaced with fresh drug-containing medium, with Incucyte detection 72 h post transduction to determine the number of GFP-positive cells. (**CandD**) Vero cells were incubated with drug as described for 1 h before addition of SV40 virions at an MOI of 1 for 2 h with occasional agitation. Fresh drug-containing medium was then added for an incubation of 24 h before fixation and permeabilisation. SV40 T-antigens were immunostained using an SV40 LT/ST antibody and species-specific Alexa Fluor 488 secondary antibody. Wells were then imaged using an Incucyte ZOOM instrument to determine the number of T-antigen positive cells, which are presented as a percentage relative to an untreated control.

It has previously been shown that verapamil, alongside a panel of classical L-type inhibitors could inhibit the entry of EBOV (Sakurai et al., 2015). Further investigation however identified that EBOV did not require L-type Ca^2+^ channel activity, with blockage of NAADP-dependent TPCs that regulate endosomal Ca^2+^ signalling, sufficient in preventing endolysosomal fusion of virus-containing vesicles with the ER. Given that PyVs traffic through the ER and verapamil treatment led to a negative phenotype independent of the assessed Ca^2+^ channel inhibitors, the importance of TPCs during MCPyV and SV40 entry was investigated. Gabapentin, an L-type Ca^2+^ channel inhibitor had no effect on MCPyV or SV40, which was comparable with nitrendipine treatment (Fig. 6A and C). However, treatment with the TPC inhibitor tetrandrine led to a striking concentration-dependent inhibition of both viruses, with near complete abolishment of fluorescent cells for MCPyV and SV40 at 5 μM and 10 μM, respectively (Fig. 6B andC). Loss of infectivity for both viruses confirmed that NAADP Ca^2+^ channels were essential for PyV infection and may represent a conserved target to restrict a wider range of PyV infections.

### 3.6 TPC inhibition prevents SV40 ER disassembly

**Fig. 7:**
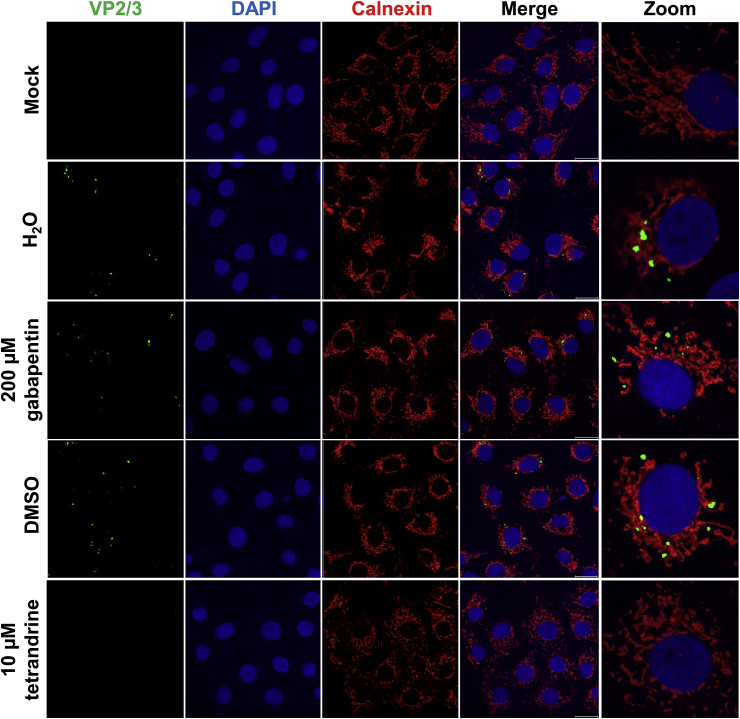
Two pore channel inhibition prevents SV40 disassembly and exposure of minor capsid proteins. Vero cells were chilled at 4 °C for 1 h before addition of SV40 virions at an MOI of 3 in chilled growth medium. Cells were maintained at 4 °C for 1 h with occasional agitation to facilitate binding. Pre-warmed growth medium containing drug was then added prior to incubation at 37 °C for 10 h before fixation. Following permeabilisation, immunostaining was performed to detect SV40 VP2/3 and the ER using calnexin. DAPI was used to visualise nucleic acids.

Although proteolytic rearrangements are initiated in acidifying endosomes, SV40 capsid disassembly sufficient for minor capsid protein exposure does not occur until the virion is processed in the ER (~6–8 hpi), with detection in the cytoplasm at 10 hpi (Kuksin and Norkin, 2012). Immunostaining of VP2/3 10 hpi to detect disassembled virions in the ER and cytoplasm displayed distinct puncta in cells treated with vehicle or gabapentin (Fig. 7). In contrast, cells treated with tetrandrine displayed no detectable puncta suggesting that the capsid was unable to disassemble and expose the minor capsid NLSs required for transit to the nucleus. These results highlight an essential requirement for NAADP-stimulated Ca^2+^ channel activity during SV40 infection.

## 4 Discussion

To date, studies regarding early events in the lifecycle of PyVs are limited. All studied PyVs traffic through the endolysosomal network during virus entry, which we confirmed for both MCPyV and SV40 using newly developed, high-throughput fluorescence-based assays (Fig. 1, Fig. 2) (Tsai and Qian, 2010). However, the specific routes of endosomal translocation and the host factors required during trafficking to the ER remain largely undefined and better understanding of this will aid in the search for antivirals. Whilst it has long been understood that the acidification of endosomes is essential for PyV entry cues, the endosomal balance of other ions and their crucial roles during the infection of a plethora of viruses is only beginning to emerge (Hover et al., 2017; Qian et al., 2009). There is a long-standing acceptance that acidification of maturing endosomes and lysosomes is due to the translocation of H^+^, which whilst true, only reflects one aspect of the highly dynamic ionic flux that regulates compartmental pH (Scott and Gruenberg, 2011). Given that ion channels regulate a wide variety of cellular functions, there is an array of well characterised pharmaceutically available drugs that can be used to treat many diseases. The ability to identify and repurpose drugs is therefore a viable and cost effective means of restricting PyV-associated diseases.

The entry of Bunyaviruses and Filoviruses have been shown to require K^+^ and Ca^2+^ channels, respectively (Gehring et al., 2014; Hover et al., 2016). Therefore TEA and verapamil were applied during attachment and entry of MCPyV and SV40. The results indicated that MCPyV required both K^+^ and Ca^2+^ channel activity, whilst SV40 trafficking was solely sensitive to Ca^2+^ channel blockage (Fig. 3). The use of a wider panel of K^+^ channel inhibitors suggested that MCPyV required the activity of K_V_ channels (Fig. 4A). Insensitivity to K^+^ channel inhibitors during SV40 infection (Fig. 4B) highlighted mechanistic differences between MCPyV and SV40. Importantly, neither blocker inhibited SV40 so their effects cannot be mediated through modulation of endosomal pH. However, further screening with other human PyVs such as JCPyV and BKPyV may identify conserved requirements that could be targeted in the treatment of PyV-associated disease in humans.

**Fig. 8:**
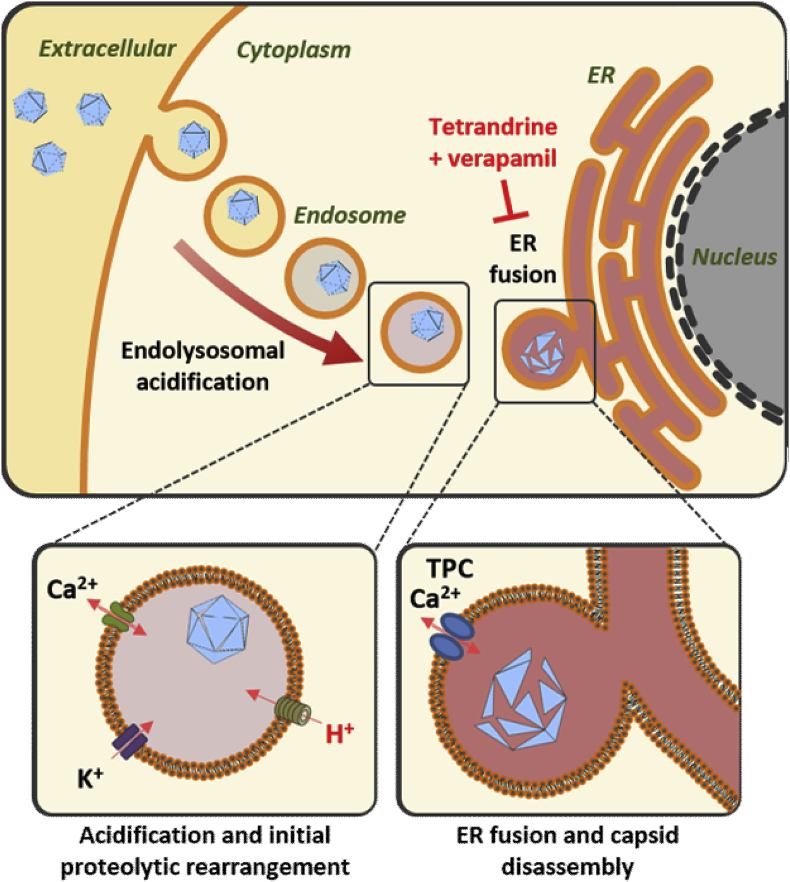
Schematic representation of proposed ion channel requirements during MCPyV and SV40 entry. Following internalisation, MCPyV and SV40 require an acidic environment. In addition to lowered pH, MCPyV also requires the activity of K^+^ and T-type Ca^2+^ channels whilst trafficking to the ER (**A**). Fusion to the ER of both MCPyV and SV40 is dependent upon NAADP-sensitive Ca^2+^ channel activity where the capsid disassembles, exposing the minor capsid proteins VP2/3 prior to translocation into the cytoplasm, which is inhibited by verapamil and tetrandrine.

Due to conserved sensitivity of MCPyV and SV40 upon challenge with verapamil, the role of Ca^2+^ channels was further explored. We selected a pharmacological approach to define the specific channel families to avoid any effects on non-conducting functions, as would be observed for gene silencing approaches. Treatment with flunarizine and nitrendipine, T- and L-type Ca^2+^ channel inhibitors, respectively, produced somewhat surprising results given that neither drug inhibited SV40 infection (Fig. 5A–D). The lack of phenotypic change for SV40 was however comparable to data relating to Ebola virus (EBOV), where verapamil was shown to prevent docking of virion-containing endosomes with the ER through inhibition of NAADP-sensitive Ca^2+^ channels (Sakurai et al., 2015). Treatment with the NAADP-sensitive Ca^2+^ channel inhibitor tetrandrine showed a concentration-dependent effect upon both viruses, with ablation of entry for MCPyV and SV40 at 5 μM and 10 μM, respectively, whereas the L-type Ca^2+^ channel inhibitor had no effect (Fig. 6A–D). As tetrandrine-mediated inhibition of EBOV entry was due to the prevention of ER docking, a viral uncoating assay for SV40 was performed as further validation (Fig. 7). Consistent with previous results, following treatment with 10 μM tetrandrine VP2/3 was undetectable, suggesting that virions either did not enter the ER or were unable to disassemble to reveal VP2/3 (summarised in Fig. 8), however it must be noted that the assay does not rule out the unlikely possibility that tetrandrine affects the rate on initial infection. Identification that PyVs share a conserved requirement with EBOV suggests that NAADP-sensitive TPC inhibition represents a therapeutic target for viruses and pathogens that transit through the ER. More recently, a similar requirement of TPC2 and inhibitory effect of tetrandrine has been identified during entry of the new coronavirus, SARS-CoV-2 (Ou et al., 2020). Although tetrandrine is not widely available and there are currently limited studies into the efficacy of treatment *in vivo*, the identification of endosomal-ER fusion as a requirement for a variety of pathogens provides a common target that could potentially be exploited (Bhagya and Chandrashekar, 2018, 2016). Further studies using knock outs of specific ion channels, particularly the NAADP-sensitive TPCs 1 and 2, as well as transient Ca^2+^ channels such as TRPML1 and TRPM2, will contribute to a better understanding of the roles that ion channels play during the entry of various PyVs.

In conclusion, we provide the first evidence that repurposing of clinically available therapeutics targeting ion channel activity are a viable method of restricting PyV infection. This study identifies that MCPyV is more sensitive to channel inhibition than SV40, with K_V_ and T-type Ca^2+^ channel inhibition restricting entry which may be applicable to other humans PyVs. Additionally we have demonstrated that the NAADP-sensitive TPC inhibitor tetrandrine is a potent inhibitor of both MCPyV and SV40. Ca^2+^ channel modulation is therefore a potential mechanism through which human PyV diseases associated with persistent infection could be modulated. Coupled with previous studies, this requirement reveals a conserved target to restrict a wider range of pathogens that transit through the ER.
